# Esophageal Peristalsis Disorders in ALS Patients with Dysphagia

**DOI:** 10.3390/brainsci10110820

**Published:** 2020-11-06

**Authors:** Jerzy Tomik, Klaudia Sowula, Mateusz Dworak, Kamila Stolcman, Małgorzata Maraj, Piotr Ceranowicz

**Affiliations:** 1 ENT Department, Faculty of Medicine, Jagiellonian University Medical College, Jakubowskiego 2, 30-688 Kraków, Poland; sowula.k@gmail.com (K.S.); dworakmateusz90@gmail.com (M.D.); kamila.stolcman@gmail.com (K.S.); 2 Department of Physiology, Faculty of Medicine, Jagiellonian University Medical College, Grzegórzecka 16, 31-531 Kraków, Poland; malgorzata.maraj@doctoral.uj.edu.pl

**Keywords:** esophageal peristalsis, amyotrophic lateral sclerosis, dysphagia, manometry, swallowing

## Abstract

To detect the variations of esophageal peristalsis in amyotrophic lateral sclerosis (ALS) patients with predominantly bulbar or predominantly pseudobulbar clinical presentation by using esophageal manometry (EM). Fifteen ALS patients with pseudobulbar clinical presentation (PBP) and 13 patients with bulbar presentation (BP), fulfilling WFN Criteria, were studied. EM was performed in all subjects using a flexible catheter with solid-state transducers. Swallowing was initiated with 5 to 10 mL of water (wet swallows) and saliva (dry swallows) and repeated at 30 s intervals. The manometric parameters were measured automatically and visualized by the computer system. The tracings were analyzed using Synectics software. In PBP patients, an increase of resting pressure value in the upper esophageal sphincter (UES) >45 mmHg, a wave-like course of resting pressure, and toothed peristaltic waves were observed. In BP patients, a low amplitude of peristaltic waves <30 mmHg (mean: 17 ± 5) was recorded, without signs of esophageal motility disturbance at onset or during progression. EM procedure allows objectively distinguishing dysphagia in ALS patients due to bulbar syndrome from the dysphagia due to pseudobulbar syndrome. It is important to identify PBP patients because of their high risk of aspiration.

## 1. Introduction

The esophageal swallowing phase begins with a temporary drop in pressure in the esophageal sphincter, which allows food to pass from the throat to the esophagus. The pressure in the sphincter then rises to 80–100 cm H_2_O to prevent air from passing from the pharynx into the esophagus. In turn, a peristaltic wave begins and travels through the body of the esophagus from the area of increased pressure to the area distally of lower pressure. This causes a characteristic pressure change called swallowing syndrome. The pressure of the wave produced in the esophagus differs from the pressure in the throat. The peristaltic wave travels along the esophagus at an average speed of 3–4 cm/s. As the wave travels through the lower esophagus, its sphincter relaxes, and the pressure in it drops, but never below gastric pressure [1].

There is primary esophageal peristalsis, which is a continuation of the pharyngeal peristaltic wave, which begins below the pharyngeal sphincter when the swallowed bite passes through it, and secondary peristalsis (secondary peristaltic waves), which can start at any height of the esophagus and purify the esophagus from the remains of food stuck to the mucosa, as well as from the refluxed gastric contents. Tertiary peristalsis consists of local contractions that do not move as swallowing waves and do not occur under physiological conditions [2].

It is well documented that dysphagia in amyotrophic lateral sclerosis (ALS) is presented with changes in the oral and pharyngeal phase of swallowing [3,4,5,6]. The occurrence of the changes in the esophageal phase of swallowing is rarely described in the literature, and there are controversial data regarding its pathology and the involvement of UES in the impaired swallowing mechanism in ALS patients. On the other hand, there are also some bases for relating the esophageal phase of swallowing in ALS patients with bulbar signs since they include degeneration of the vagus nerve, especially the nucleus ambiguus, from which the nerve fibers go down and innervate the striated muscle of the upper part of the esophagus. Additionally, esophageal disturbances are commonly manifested by pharyngeal or even oropharyngeal symptoms [7,8].

Symptoms of bulbar dysfunction at ALS onset can be observed in about 30% of patients, whereas 80–90% of ALS patients develop bulbar symptoms by the time of their death [3,9]. Patients suffering from ALS often develop swallowing disorders, which increase the risk of aspiration pneumonia and impair their nutritional status [3,10,11]. Dysphagia and related aspiration pneumonia are major complications that negatively affect the quality of life of ALS patients [12,13].

It has been well documented in the literature that the main cause of disturbance in the oropharyngeal phase of swallowing in ALS patients is the combination of bulbar spasticity, flaccid muscle weakness, and wasting [14,15,16,17,18].

Detection and measurement of the esophageal stage of swallowing, with an emphasis on the evaluation of the upper esophageal sphincter (UES) and esophageal body functions, can be of great importance for the complete understanding of the pathophysiology of neurogenic dysphagia as well as the mechanism of aspiration [19,20].

## 2. Material and Methods

### 2.1. Subjects

The study included 28 ALS patients (10 men and 18 women) with clinical evidence of dysphagia. Dysphagia was assessed according to the ALS swallowing severity scale (ALSSSS), which is a 10-point scale comprising a part of the ALSSSS used to evaluate symptoms of swallowing in ALS patients [15]. These patients had ALSSSS scores ranging from five to eight points. The research was carried out over a period of two years. Initially, the study included a group of 42 patients with swallowing disorders; however, at enrolment for the experiment, five people had dropped out, four people had died, and the general condition of five patients had deteriorated, which in keeping with the underlying exclusion criteria made them not eligible for the study. ALS was diagnosed in the out-patient clinic for patients with ALS affiliated with the Department of Neurology, Jagiellonian University Medical College, Krakow. The diagnosis of ALS was established by the same neurologist (Barbara Tomik) according to the EL Escorial criteria [21].

The swallowing phase disturbance was compared among ALS patients assigned to groups according to predominant symptoms. The studied patients presented a different degree of dysphagia, but none of them required nasogastric tube feeding nor percutaneous endoscopic gastrostomy. All patients presented with bulbar and pseudobulbar symptoms, such as emotional liability, reduced tongue mobility, tongue muscular atrophy and fasciculations, brisk jaw jerk, palatal and pharyngeal reflexes, spastic/flaccid/mixed dysarthria, and dysphagia. The patients were divided into groups based on the predominance of pseudobulbar (PBP) or bulbar (BP) symptoms in the neurological examination.

In the PBP group, there were 15 people: 10 women and 5 men; the mean age of patients was 56.3 ± 12.3 years. Mean values in the ALS Swallowing Severity Scale (ALSSSS) [22] were: 6.8 ± 3.1 points (7–5 range), and mean duration of dysphagia from onset to time of analysis was 8.5 ± 4.4 months (range: 2–21). Mean disease duration was measured from the presentation of first symptoms to the date of inclusion in the study—was 6–61 months (mean: 15.5 ± 11.9).

In the BP group, there were 13 patients: 8 women and 5 men; the mean age of patients at disease onset was 63.0 ± 6.7 years. Mean values in the ALSSSS were: 6.7 ± 1.9 (7–5 range), mean duration of dysphagia was correspondingly: 7.9 ± 3.7 months (range: 3–19). Mean disease duration from the occurrence of first symptoms to the date of inclusion in the study was 7–70 months (mean: 16.4 ± 12.3).

The Control group for the manometric studies consisted of 20 sex- and age-matched healthy volunteers who declared no swallowing problems (11 men and 9 women) at the mean age of 48.0 ± 12.3 years (range: 35–65 years).

Study protocol was approved by the Bioethical Committee of Jagiellonian University (KBET/123/B/2004). All subjects provided their informed consent to participate in the study.

Criteria excluding patients from the study were as follows:

1. Age below 30 years and above 75 years
2. Significant deterioration of the swallowing function in the medical history and physical examination (a score of less than five points on the ALSSSS);
3. Deterioration of the clinical status of a patient during the course of the study (i.e., a significant increase in bulbar symptoms, emergence of subjective respiratory dysfunction, cachexia, or intensification of limb symptoms making the patient unable to turn up for the study);
4. FVC (forced vital capacity) <50%, measured obligatorily by spirometry both in the seated and standing positions.
5. Anatomical changes in the oral cavity, pharynx, or esophagus that prevent objective assessment;
6. A swallowing disorder which is not the consequence of the primary disease (e.g., patients after surgical operations on the neck, local radiotherapy, or other causes of esophageal peristalsis disturbance);
7. Taking medication which negatively affects motor functions of the esophagus (e.g., calcium blockers, beta-blockers, and nitrates);
8. Comorbidities such as cardiovascular disease, especially cardiac arrhythmia, as well as other digestive tract disorders;
9. Lack of the patient’s consent to take part in the study.

### 2.2. Methods

Manometric examination of the upper part of the gastrointestinal tract was performed with the use of a computerized Synectics System (Sweden) consisting of Polygrapf VIII, a solid-state catheter Küngsberg Instrument Pasadena USA, number P 33-4200 DM60, and software Polygram Upper GI Edition, version 5.06 (Synectics, Synectics Medical AB, Stockholm, Sweden). The tests were carried out over a period of three years with the use of “solid-state” manometry because at that time, we did not have high-resolution manometers (HRM). Bearing in mind the objectivity of the conducted research, we did not intentionally change their methodology.

The study was performed in the sitting position according to the technique described by Castell and Castell [23]. The flexible esophageal catheter with solid-state transducers was positioned into the esophagus transnasally.

Esophageal pressure values were estimated directly by the solid-state transducers in the catheter. Four manometric transducers were located in the distal part of the catheter with a distance between each other of 3 cm, 2 cm, and 5 cm, respectively. The proximal part of the esophagus was evaluated by locating the proximal transducer 1 cm below the distal border of the upper esophageal sphincter (UES). The most important observations were collected from the distal probe with a possibility of circumferential measuring.

Each subject performed five dry swallowing maneuvers (saliva only) and five wet swallowing maneuvers (about 10 mL of water per each swallowing act). All patients with dysphagia presented individual rhythm of successful swallowing, which determined the total time of examination.

Before the insertion of the catheter, each patient was in the sitting position in front of the computer screen. After catheter insertion, the patient had a 5 min rest for habituation and better tolerance of the catheter presence. During this period, patients were observed in real-time their manometric changes of saliva swallowing, cough, and breathing, without the examiner’s assistance.

The following parameters were measured automatically by the computer system (Synectics, Sweden): 1, upper esophageal contractile amplitude; 2, upper esophageal contractile duration; 3, upper esophageal contractile velocity; and 4, mean value of the resting pressure in the UES in the esophageal body.

The esophageal peristalsis assessment site has been narrowed to the upper esophageal sphincter and the upper esophagus only, due to the fact that only the sphincter and the upper third of the esophageal body are composed of striated muscles that undergo neurodegeneration in ALS. But the mean negative pressure in the esophageal body, the type of peristaltic wave, the frequency of the primary and secondary peristaltic waves, and the presence of non-peristaltic waves was also assessed.

### 2.3. Statistical Analysis

The qualitative variables were expressed as counts and percentages, whereas the quantitative ones were expressed as mean and standard deviation as well as minimum and maximum values. The relationship between two qualitative variables was assessed using Pearson’s Chi-square independence test. A comparison of the quantitative variable between the two groups was conducted using the Student’s t-test for independent samples for normally distributed variables and a non-parametric Mann-Whitney-Wilcoxon test in the other case. Statistical analysis was done using STATISTICA v. 8.0. *P*-value ≤ 0.05 was regarded as statistically significant.

## 3. Results

There were no significant differences between age and sex in the studied group. The mean value of the Norris scale was 72.7 ± 27.2 points (range: 46–101). The mean value of ALS Functional Rating Scale Revised (ALSFRS-R) [24] was 22.9 ± 8.5 points (range: 16–37). The mean value of the clinical scale of swallowing was 6.2 ± 1.6 points (range: 5–7 points). The mean duration of dysphagia was 8.6 ± 6.4 months (range: 1–26).

The UES resting pressure was 33 ± 6.2 and 34 ± 5.7 mmHg in ALS and 30 ± 7.8 and 31 ± 6.3 mmHg in the control group during dry and wet swallows, respectively. The UES relaxation was complete with the reduction of the resting pressure by more than 5 mmHg in all the healthy controls but decreased in ALS patients. We did not observe the UES relaxation in six ALS patients during dry (Table 1) and in four ALS patients during wet swallows (Table 2).

The mean value of the intraesophageal pressure was −1 ± 0.8 and −1 ± 0.9 mmHg in ALS patients and −6 ± 1.9 and −6 ± 2.3 mmHg in the control group (*p* = 0.007), during dry and wet swallows, respectively.

Comparison of the following parameters was conducted: mean contractile amplitude of UES and the upper part of esophageal body, contractile duration, velocity, mean negative pressure values in esophageal body, reduced wave propagation, the type of peristaltic waves, and mean values of resting pressure in UES in wet and dry swallows, in patients from the group with the predominance of bulbar symptoms (BP) and those with mainly pseudobulbar symptoms (Table 3).

**Figure 1:**
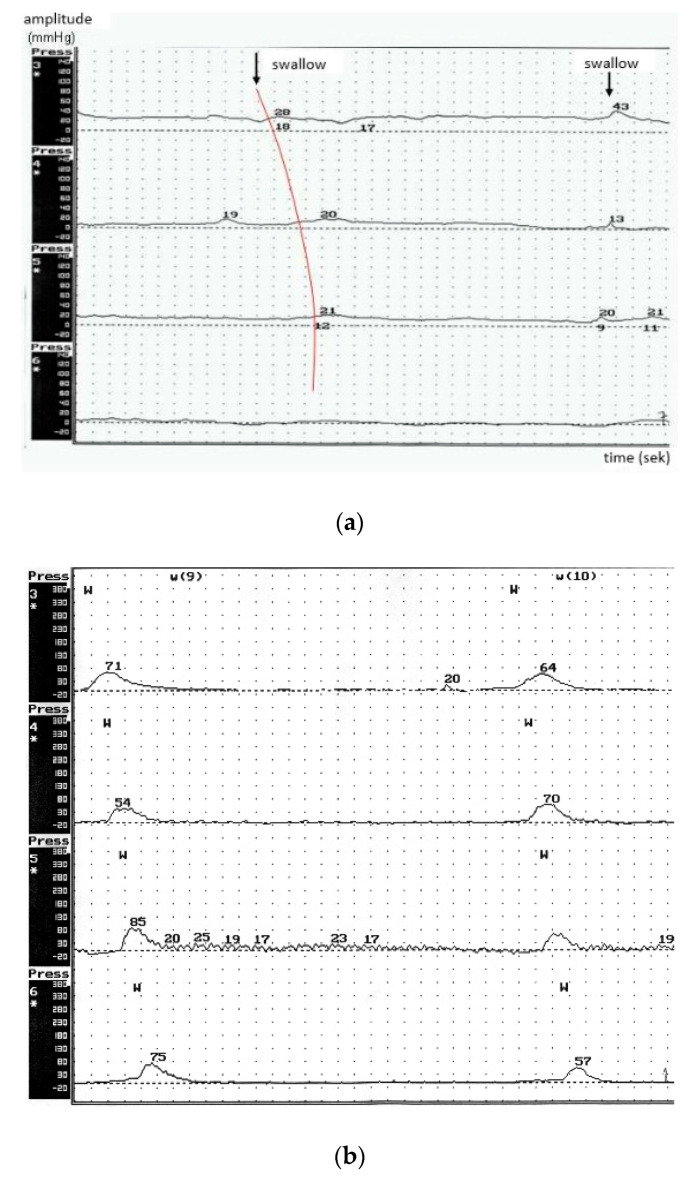
(**a**) Manometric study results of the esophagus in ALS patients with predominance of bulbar symptoms—advanced dysphagia. (**b**) Manometric study results of the esophagus in healthy controls.

To conclude, in the BP group a low (<40 mm/Hg) mean amplitude of peristaltic waves was reported without signs of peristaltic function of the esophagus (Figure 1a). Manometric study results of the esophagus in healthy controls are shown in Figure 1b. No propulsive peristalsis was recorded despite stimulation by the oral swallowing phase or positive intraesophageal pressure. Decreased motility of the esophagus represents its hypotonic dysfunction.

**Figure 2:**
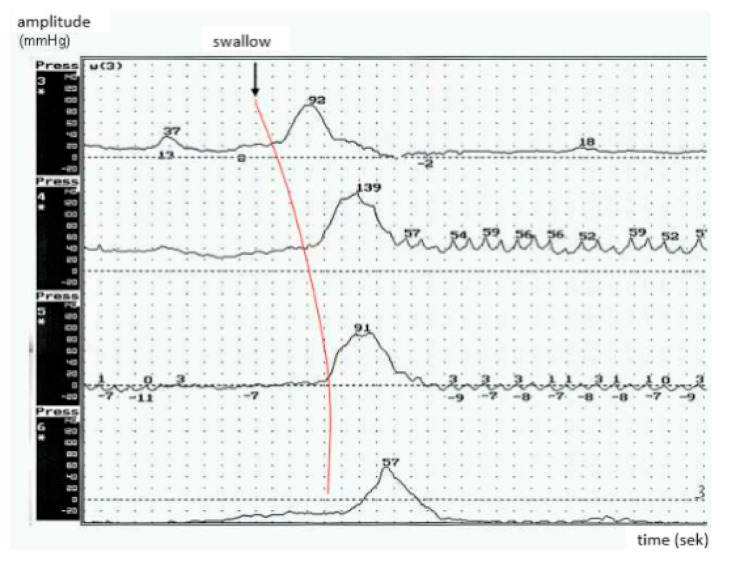
Esophageal peristalsis dysfunction in ALS patients with the predominance of pseudobulbar syndrome and dysphagia.

In the PBP group, an increase (>45 mmHg) of resting UES pressure, a wave-like course of resting pressure, and toothed peristaltic waves were observed (Figure 2). The figure represents dysfunctional esophageal body peristalsis—inadequate maximum pressure for each peristaltic wave in the proximal part of the esophagus (striated muscles), reduced wave velocity, and their disturbed propagation to the distal part of the esophagus (a hypertonic dysfunction).

Based on the analysis of manometric data obtained from ALS patients assigned to BP and PBP group, three types of peristalsis dysfunctions in the esophageal body were recognized:

### 3.1. Type 1

**Figure 3:**
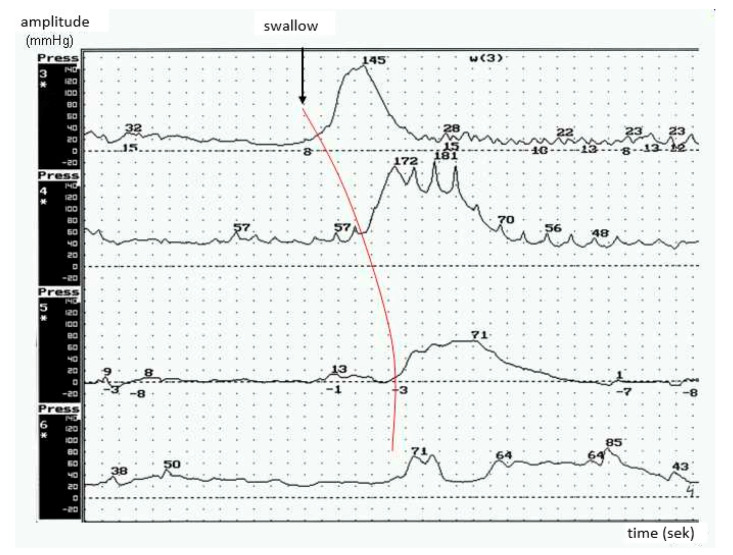
ALS-hypertonic esophageal body peristalsis with high amplitude of peristaltic waves (patients with the predominant pseudobulbar syndrome).

Hypertonic dysfunction was detected in 12 out of 28 patients. Within this group, all of the patients presented with pseudobulbar symptoms. The mean values of the resting pressure of UES were above 40 mmHg; the course of the resting pressure was wave-like; the peaks of peristaltic waves were acute (Figure 3).

### 3.2. Type 2

**Figure 4:**
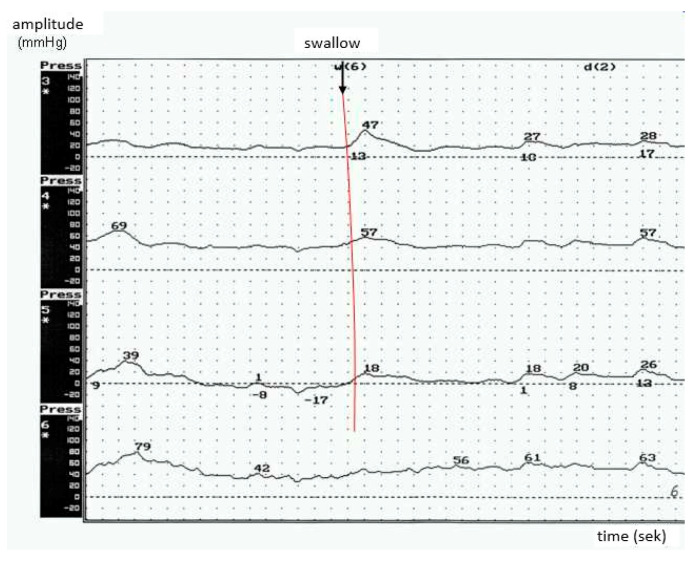
Hypotonic type of esophageal body peristalsis in ALS-low amplitude of peristaltic waves (patients with predominant bulbar syndrome).

Hypotonic dysfunction was detected in 13 out of 28 patients. Within this group, all of the patients were characterized by the predominance of bulbar symptoms. The mean amplitude of peristaltic waves was <30 mmHg, and no esophageal dysmotility was recorded. Adaptive relaxation in the esophageal body during the primary peristaltic waves was observed, as well as a high number of non-peristaltic waves (Figure 4).

### 3.3. Type 3

**Figure 5:**
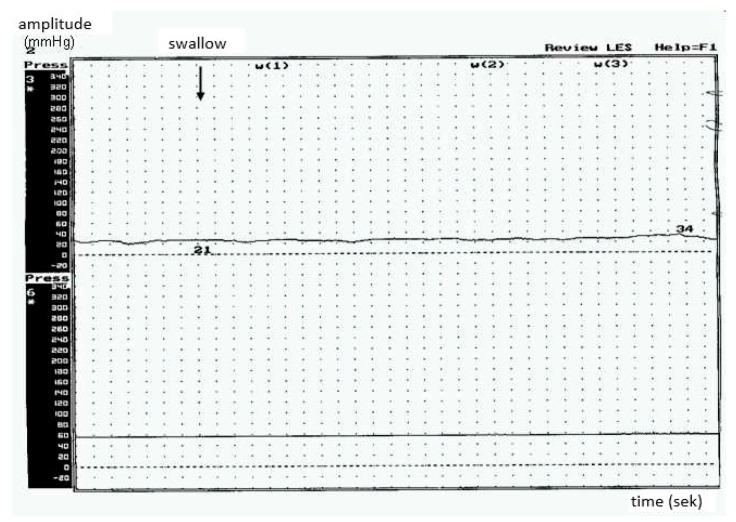
Atonic ALS of esophageal body peristalsis—no peristaltic waves were recorded (patients with bulbar ALS).

Atonic dysfunction (so called “decanting” type) occurred in 3 out of 28 patients. In this group the neurological examination revealed more pronounced bulbar dysfunction with the upper palate palsy and anarthria. The manometric examination recorded no peristaltic waves, neither primary, nor secondary throughout the esophageal body. No adaptive relaxation was observed, nor the presence of central swallowing reflexes in the esophageal body. Low esophageal pressure was accompanied by moderate symptoms of dysphagia associated with increased sensitivity of the esophagus and the swallowing in the “decanting” mechanism (Figure 5).

## 4. Discussion

In the present study, esophageal peristalsis was estimated in 28 ALS patients with mild or moderate dysphagia by using esophageal manometry and compared with the results obtained from the control subjects. We found strong evidence of the occurrence of disturbances of the esophageal body in ALS dysphagic patients. The detailed analysis showed that the parameters of the upper esophageal body peristalsis, e.g., the mean esophageal contractile amplitude, duration, and velocity, were significantly different during the wet and dry swallows in the ALS patients as compared to the controls. Also, the result differences between the parameters obtained during the wet and dry swallows in the studied groups were statistically significant only for the amplitude and the velocity in the controls (*p* < 0.001) but not in the ALS subjects. However, we also observed no statistical association between the values of the mean esophageal body contractile amplitude, duration, and velocity during the wet and also dry swallows (compared separately) and the mean values of clinical scales assessing the disease severity (the Norris scale and ALSFRS), as well as the mean values of the clinical scale of swallowing.

In clinical practice, there are no “pure” syndromes of damage to only the upper or only the lower motor neuron at the level of the brainstem in patients with ALS (except for symptoms from the upper motor neuron at the level of the bulb in patients diagnosed with primary lateral sclerosis). These types of isolated symptoms in ALS may appear as harbingers of disease at the bulbar level. They are accompanied by symptoms of damage to the second motor neuron as the disease progresses. An experienced clinician is able to assess the prevalence of clinical symptoms from one motor neuron in the clinical picture. Such a division of ALS patients with bulbar symptoms is encountered in the literature, especially when assessing dysphagia in this disease [3]. The division of patients with ALS into patients with bulbar and pseudo-bulbous forms allows for a more accurate assessment of disorders of the oropharyngeal and esophageal phases of swallowing in these two groups. Determining the profile of manometric disorders (syndromes of esophageal motility disorders) and the values of manometric parameters for each of these groups may be useful in the diagnosis of patients with ALS in terms of the possible increased risk of food aspiration to the respiratory tract.

No significant differences between the wet and dry swallows in the ALS patients were observed. This phenomenon demonstrates the absence of specific swallowing reflexes induced by saliva or water. Dry swallows in the ALS patients with a long duration of contraction (4.8 ± 1.9 s) and higher velocity (5.4 ± 2.0 cm/s) explain the difficulties in saliva swallowing reported by the patients. Of course, these symptoms may also be caused by oropharyngeal dysmotility and sialorrhea.

Our data suggest that the neurodegeneration of cortical and bulbar motor neurons in ALS patients is parallel to the esophageal motility abnormalities observed. The process of denervation probably leads to the disruption of neural reflexes controlling the swallowing and limits the effectiveness of the primary esophageal peristalsis. The number of primary swallows was lower in all ALS patients than in the controls. Central denervation and reduced mechanical stimulation of the striated muscle in the oesophageal wall can lead to atrophic changes, which may aggravate dysphagia [25].

There are conflicting data regarding the manometric changes of UES in ALS patients. It is known that decreasing resistance of UES contributes to the relief of dysphagia, and cricopharyngeal myotomy has been performed in ALS patients [26,27]. Incomplete UES relaxation or, sometimes, the elevation of UES pressure during bolus passage (UES spasm) inevitably results in dysphagia, probably because of vagus nerve involvement in ALS neurodegeneration [28]. UES has so far been considered a cause of aspiration in ALS; however, the occurrence of the UES spasm in ALS is still under discussion. MacDougall et al. [29], using the pharyngoesophageal manometry in the small group of 13 ALS patients and 13 controls, showed a significant reduction of the post-contraction amplitude of UES during wet and solid swallows in 7 (out of 13) ALS patients with severe dysphagia, which was not observed in the controls. Kawai et al. [30], also using the pharyngoesophageal manometry in 11 ALS patients, showed that manometric parameters were almost normal in all cases except one; however, it must be noted that the study included ALS patients with the early stage of dysphagia. Considering the above data, the UES spasm did not contribute to dysphagia in the bulbar ALS cases. On the other hand, Higo et al. [28] have studied, by videomanofluorometry, the time-course changes of the swallowing function in 21 ALS patients and showed the incomplete relaxation of UES in 4 patients, the elevation of UES pressure during the bolus passage in 3 cases, as well as more cases of aspiration (57.1%) in ALS patients with the UES spasm. However, it was found out that elevation of the larynx and UES opening were relatively well-maintained in ALS patients until one year from onset of bulbar symptoms [31].

We did not observe any case of UES spasm: the mean resting pressure of UES was lower than 45 mmHg in all the dysphagic patients. However, the UES relaxation was incomplete (reduction of resting pressure <5 mmHg) in four patients during wet and in six patients during dry swallows. We demonstrated significant abnormalities of all measured parameters of the upper oesophageal body peristalsis in all the dysphagic ALS patients as compared to the controls.

The presented results indicate a more pronounced dysfunction of the swallowing phase in ALS patients with the predominance of pseudobulbar syndrome (which can be referred to as “esophageal spasticity”); therefore, corticobulbar pathways degeneration may be responsible for the impaired swallowing, what has been reflected in literature. In ALS, the degeneration of corticobulbar pyramidal fibers and central denervation can be observed; however, the involuntary swallowing mechanism is preserved as it remains under the control of the bulbar swallowing center. In ALS, any abnormalities in the voluntary swallowing act can be attributed to the degeneration of pyramidal cortico-bulbar and central control dysfunction. The delay in swallowing reflexes after voluntary wet or dry swallows is related to the progression of excitatory corticobulbar fibers degeneration. The loss of corticobulbar control makes all swallows spontaneous (involuntary) and subject to the control of the bulbar swallowing center [9,32]. The observation that dysphagia occurs more frequently and is more pronounced in patients with more substantial damage of corticobulbar fibers has been confirmed by other authors [2,14,32].

Adequate velocity and effectiveness of UES relaxation lead to its opening, and the bolus passes to the esophagus. As a result of UES opening, the pressure in the esophageal lumen decreases and creates suction force from the laryngopharynx.

In ALS patients with symptoms of pseudobulbar syndrome, UES opening dysfunctions are caused by its over-reactivity and increased spasticity [3,28]. The inadequate mechanism of UES opening and closing in ALS is most surely the result of increased reflex reactivity hyper reflexive hypertonic cricopharyngeal muscle caused by the loss of its central inhibition [1]. The described UES activity seems justified when taking into account the frequent increase of jaw jerk and other brainstem reflexes such as palatal and pharyngeal reflexes. Hughes and Wiles [33] prove that the loss of corticobulbar fibers leads to exaggerated palatal and pharyngeal reflexes in ALS because of the reduction of the efferent inhibitory fibers and it can be responsible for the impaired swallowing. Also, Hadjikoutis and Wiles [34] point to the occurrence of overly pronounced reflexes of pharyngeal muscles next to exaggerated deep tendon reflexes in ALS, which is associated with increased swallowing disturbance and the necessity to reduce the volume of fluids and the size of food bites. Because as ALS progresses, dysphagia gets worse due to the weakening of pharyngeal musculature, there are reports in which measured a pharyngeal pressure using high-resolution manometry [35].

Moreover, the pseudobulbar damage is characterized by the dysfunction between voluntary and involuntary swallowing mechanisms, such as the absence of palate elevation during phonation with exaggerated palatal and pharyngeal reflexes [4]. On the other hand, it has been known that the proper function of the palate is a key element for generating adequate pressure inside the oral cavity and negative pressure during the pharyngeal phase of swallowing [4,36]. The damage of upper motor neuron (pseudobulbar syndrome) and the weakening of the tongue and the floor of mouth muscles are important elements of the swallowing pathomechanism initiated voluntarily, whereas in patients with lower motor neuron damage (bulbar syndrome), both the voluntary and autonomic movement is weakened [3].

There are reports that significant HRM parameters highly specific for the type of nutrition and the possibility of oral feeding in ALS have been identified.

## 5. Conclusions and Future Directions

We propose the use of EM as an objective, manometric method for the detection of esophageal peristalsis failure in neurogenic dysphagia. We have shown that in our patients with ALS dysphagia using esophageal manometry, this method allows us to determine the type of peristalsis disorders in patients with ALS due to bulbar syndrome and patients with pseudobulbar syndrome. Drawing on these data, we suggest the coexistence of the disturbances of the oropharyngeal phase of swallowing, which is well documented in ALS patients, also with changes in the upper esophageal peristalsis.

## Figures and Tables

**Table 1:** Comparison of mean resting pressure values in upper esophageal sphincter (UES), mean pressure values in esophageal body, mean amplitude, contractile duration, and velocity during dry swallows in dysphagic amyotrophic lateral sclerosis (ALS) patients and the control group.

	ALS	Control Group	Statistical Significance *p*
**Mean resting pressure of UES (mmHg)**	33 ± 6.2	30 ± 7.8	NS **
**Number of patients with incomplete relaxation of UES**	6	0	NS **
Mean contractile amplitude * (mmHg)	96 ± 59	44 ± 25	*p* < 0.001
Mean contractile duration * (s)	4.8 ± 1.9	2.5 ± 1.7	*p* < 0.001
Mean contractile velocity * (cm/s)	5.4 ± 2.0	3.9 ± 0.5	*p* = 0.002
Mean negative pressure in esophageal body *	−1 ± 0.8	−6 ± 1.9	*p* = 0.007

* Esophageal body: 3 cm below the UES (upper esophageal sphincter); NS **—not significant.

**Table 2:** Comparison of mean resting pressure values in upper esophageal sphincter (UES), mean pressure values in the esophageal body, mean amplitude, contractile duration, and velocity during wet swallows in dysphagic ALS patients and the control group.

	ALS	Control Group	Statistical Significance *p*
**Mean resting pressure of UES (mmHg)**	34 ± 5.7	31 ± 6.3	NS **
**Number of patients with incomplete relaxation of UES**	4	0	NS **
Mean contractile amplitude * (mmHg)	99 ± 58	60 ± 22	*p* = 0.009
Mean contractile duration * (s)	3.91 ± 1.4	2.7 ± 0.7	*p* = 0.001
Mean contractile velocity *(cm/s)	4.6 ± 1.8	2.8 ± 0.4	*p* < 0.001
Mean negative pressure in esophageal body *	−1 ± 0.9	−6 ± 2.3	*p* = 0.007

* Esophageal body: 3 cm below UES; NS **—not significant.

**Table 3:** Results obtained from the manometric examination of the esophagus in ALS patients with predominance of bulbar (BP) or pseudobulbar symptoms (PBP).

Measured Parameter	BP (*n* = 13)	PBP (*n* = 15)
**Contractile amplitude**	max: 38.6 ± 6 mmHg mean: 17 ± 5	max: 98.6 ± 19 mmHg mean: 67 ± 15
**Mean contractile duration**	1.4 ± 0.2 s	5.2 ± 1.5 s
**Negative pressure in the esophageal body**	in all patients	in 3 out of 15 patients
**Reduced wave propagation**	in 8 out of 13 patients	in 3 out of 15 patients
**Peristaltic waves velocity**	2 cm/s	4.3 cm/s
**Type of peristaltic waves**	Regular, parabolic	Irregular
**Mean resting pressure of UES**	12 ± 2.4 mmHg	55 ± 17 mmHg
**Number of effective swallows**	3–5/min.	Non-decanting type: 6–9/min. Decanting type: difficult to determine, low amplitude of pressures
